# A Revisit to the Research Updates of Drugs, Vaccines, and Bioinformatics Approaches in Combating COVID-19 Pandemic

**DOI:** 10.3389/fmolb.2020.585899

**Published:** 2021-01-25

**Authors:** Tofael Ahmed Sumon, Md. Ashraf Hussain, Md. Tawheed Hasan, Mahmudul Hasan, Won Je Jang, Eleus Hussain Bhuiya, Abdullah Al Mamun Chowdhury, S. M. Sharifuzzaman, Christopher Lyon Brown, Hyun-Ju Kwon, Eun-Woo Lee

**Affiliations:** ^1^Department of Fish Health Management, Sylhet Agricultural University, Sylhet, Bangladesh; ^2^Department of Fisheries Technology and Quality Control, Sylhet Agricultural University, Sylhet, Bangladesh; ^3^Department of Aquaculture, Sylhet Agricultural University, Sylhet, Bangladesh; ^4^Department of Pharmaceuticals and Industrial Biotechnology, Sylhet Agricultural University, Sylhet, Bangladesh; ^5^Department of Biotechnology, Pukyong National University, Busan, South Korea; ^6^Division of Analytical Development, Globe Biotech Limited, Dhaka, Bangladesh; ^7^Department of Surgery, Rangamati General Hospital, Rangamati, Bangladesh; ^8^Institute of Marine Sciences, University of Chittagong, Chittagong, Bangladesh; ^9^World Fisheries University Pilot Programme, Pukyong National University, Busan, South Korea; ^10^Biopharmaceutical Engineering Major, Division of Applied Bioengineering, Dong-Eui University, Busan, South Korea

**Keywords:** COVID-19, SARS-CoV-2, drugs, vaccines, *in silico* approaches

## Abstract

A new strain of coronavirus severe acute respiratory syndrome coronavirus 2 (SARS-CoV-2) responsible for the coronavirus disease 2019 (COVID-19) pandemic was first detected in the city of Wuhan in Hubei province, China in late December 2019. To date, more than 1 million deaths and nearly 57 million confirmed cases have been recorded across 220 countries due to COVID-19, which is the greatest threat to global public health in our time. Although SARS-CoV-2 is genetically similar to other coronaviruses, i.e., SARS and Middle East respiratory syndrome coronavirus (MERS*-*CoV), no confirmed therapeutics are yet available against COVID-19, and governments, scientists, and pharmaceutical companies worldwide are working together in search for effective drugs and vaccines. Repurposing of relevant therapies, developing vaccines, and using bioinformatics to identify potential drug targets are strongly in focus to combat COVID-19. This review deals with the pathogenesis of COVID-19 and its clinical symptoms in humans including the most recent updates on candidate drugs and vaccines. Potential drugs (remdesivir, hydroxychloroquine, azithromycin, dexamethasone) and vaccines [mRNA-1273; measles, mumps and rubella (MMR), bacille Calmette-Guérin (BCG)] in human clinical trials are discussed with their composition, dosage, mode of action, and possible release dates according to the trial register of US National Library of Medicines (clinicaltrials.gov), European Union (clinicaltrialsregister.eu), and Chinese Clinical Trial Registry (chictr.org.cn) website. Moreover, recent reports on *in silico* approaches like molecular docking, molecular dynamics simulations, network-based identification, and homology modeling are included, toward repurposing strategies for the use of already approved drugs against newly emerged pathogens. Limitations of effectiveness, side effects, and safety issues of each approach are also highlighted. This review should be useful for the researchers working to find out an effective strategy for defeating SARS-CoV-2.

## Introduction

The novel coronavirus disease, COVID-19, was first identified in the city of Wuhan, China at the end of December 2019. At the onset of the disease a series of pneumonia incidents were reported to China National Health Commission on 7 January 2020. Subsequently, similar cases spread rapidly throughout the world, and the World Health Organization (WHO) declared the situation a global pandemic on 11 March 2020 (Tahir ul Qamar et al., 2020a; Wang D. et al., 2020). As of 22 November 2020, around 57 million confirmed cases and over 1.3 million deaths have been reported in 220 countries and territories across the world (WHO, 2020b). The causative agent of COVID-19 is named severe acute respiratory syndrome (SARS)-CoV-2 by the International Committee on Taxonomy of Viruses due to 89% nucleotide similarity with bat SARS-like CoVZXC21 and 82% with human SARS-CoV (Abd El-Aziz and Stockand, 2020; Chan et al., 2020).

To prevent loss of lives and socioeconomic impacts due to COVID-19, scientists are currently undertaking numerous trials to find preventive measures and therapeutics to control the pandemic at the earliest possible time. As of 22 November 2020, around 4,000 studies on COVID-19 were registered in the US National Library of Medicine (NLM) website^1^, of which many are ongoing in different hospitals around the world. These studies mostly focused on vaccines trials, drugs development, and *in silico* therapeutics for the patients.

Clinical trials of antiviral drugs, such as remdesivir (Beigel et al., 2020), hydroxychloroquine and azithromycin (Gautret et al., 2020), favipiravir (Chen C. et al., 2020), ritonavir and lopinavir (Hung et al., 2020), methylprednisolone, epoprostenol, sirolimus, sarilumab, and anakinra (Wu R. et al., 2020) are ongoing in China, US, UK, and several European countries. Among them, remdesivir is effective against CoVs related to SARS, MERS (Amanat and Krammer, 2020), and Ebola virus, although comparatively less effective than other treatments (Mulangu et al., 2019). Likewise, chloroquine and hydroxychloroquine, which promote antiviral actions against human immunodeficiency virus (HIV) and acquired immune deficiency syndrome (AIDS), are on trial to treat COVID-19 patients (Rosa and Santos, 2020). Moreover, lopinavir, ritonavir, arbidol, and favipiravir are under trial phases all over the world, but their efficacy is yet to be confirmed, and some of the trials have been terminated due to failure in patients^1^.

There are 16 vaccines in human trials (biorender.com), including some that have been used previously and patented. Owing to the genetic similarities, previously developed SARS and Middle East Respiratory Syndrome (MERS) vaccines might be effective (Liu C. et al., 2020), but their clinical trials against SARS-CoV-2 infection are yet to take place. WHO has accorded many vaccines based on a variety of technologies, and only RNA and non-replicating vector vaccines are brought into human safety trials. Although a few vaccines (mRNA-1273, ChADOx1 nCoV-19, MMR) have entered into their third and fourth trial phases and thousands of volunteers have been recruited, thus far, none are confirmed to be operative against COVID-19 (Cohen, 2020b).

Researchers have suggested the use of some acknowledged antiviral drugs like nucleoside analogs, RNA-dependent RNA polymerase (RdRp), HIV protease inhibitors, and angiotensin-converting enzyme 2 (ACE2) as promising for COVID-19 treatment (Shah et al., 2020). For instance, three CoV-2 chimeric proteins nucleocapsid, ORF3a, and membrane proteins are evaluated by docking models and constructed a multiepitope vaccine candidate NOM, which is capable of modulating humoral and cell-mediated immune responses (Enayatkhani et al., 2020). In addition, statins, a group of cholesterol-lowering drugs known to inhibit the enzyme SARS-CoV-2 main protease (Mpro), could be a potential drug target. Reiner and collaborators demonstrated that statins (pitavastatin, rosuvastatin, lovastatin, and fluvastatin) hold the binding energy to inhibit SARS-CoV-2 Mpro (Reiner et al., 2020). Moreover, a number of *in silico* studies revealed that peptide-like and small molecules including drugs (cobicistat, ritonavir, lopinavir, and darunavir) are potentially effective CoV-2 protease inhibitors (Pant et al., 2020; Shah et al., 2020). Added to this, some non-traditional drug discovery techniques, such as artificial intelligence (AI) and machine learning, showed potential to develop alternative treatments and therapeutics for COVID-19 (Omolo et al., 2020).

Researchers from various locations are seeking therapeutics for the prevention and control of COVID-19. Previously, limited reviews like vaccine pipeline of SARS-CoV-2 (Abd El-Aziz and Stockand, 2020; Chan et al., 2020) and clinical features of COVID-19 patients (Huang C. et al., 2020) were published to provide frequent updates about CoV-2. There is no report that included *in silico* approaches related to studies on drugs and vaccines against COVID-19. The present study comprehensively reviewed recent literature on various drugs, vaccines, and computational bioinformatics approaches relevant to COVID-19. A revisit to the discoveries of COVID-19 therapeutics is intended to provide updated knowledge about ongoing trials and future scope for investigation, of interest to researchers, and policymakers.

## Methodology and Data Collection Approaches

This review article is written on the basis of selected evidence from the literature available on Google Scholar and PubMed published in reputable journals. The criteria considered for searching articles on the web are the key words like SARS-CoV-2, COVID-19, drugs, vaccines, *in silico* approaches, drug suggestions for COVID-19, clinical trials of the drugs and vaccines, etc., and publication date and journal impact were also considered. In most cases, articles published in 2020 and during the COVID-19 period were taken into account. A few papers published before 2020 and websites updating situation reports are also cited here to document previous viral outbreaks. The health register of the US National Library of Medicine (clinicaltrials.gov), European Union (clinicaltrialsregister.eu), Chinese Clinical Trial Registry (chictr.org.cn), and Vaccine Tracker (biorender.com) were emphasized for their consideration of trials of drugs and vaccines worldwide. All published articles including some preprints from *aRxiv* and *medRxiv* are extensively reviewed and cited. Therefore, this review paper provided a broad and shallow overview of the research landscape of COVID-19 pandemic that could be useful for background information of the topic.

## Pathogenesis and Symptoms of COVID-19

**Figure 1:**
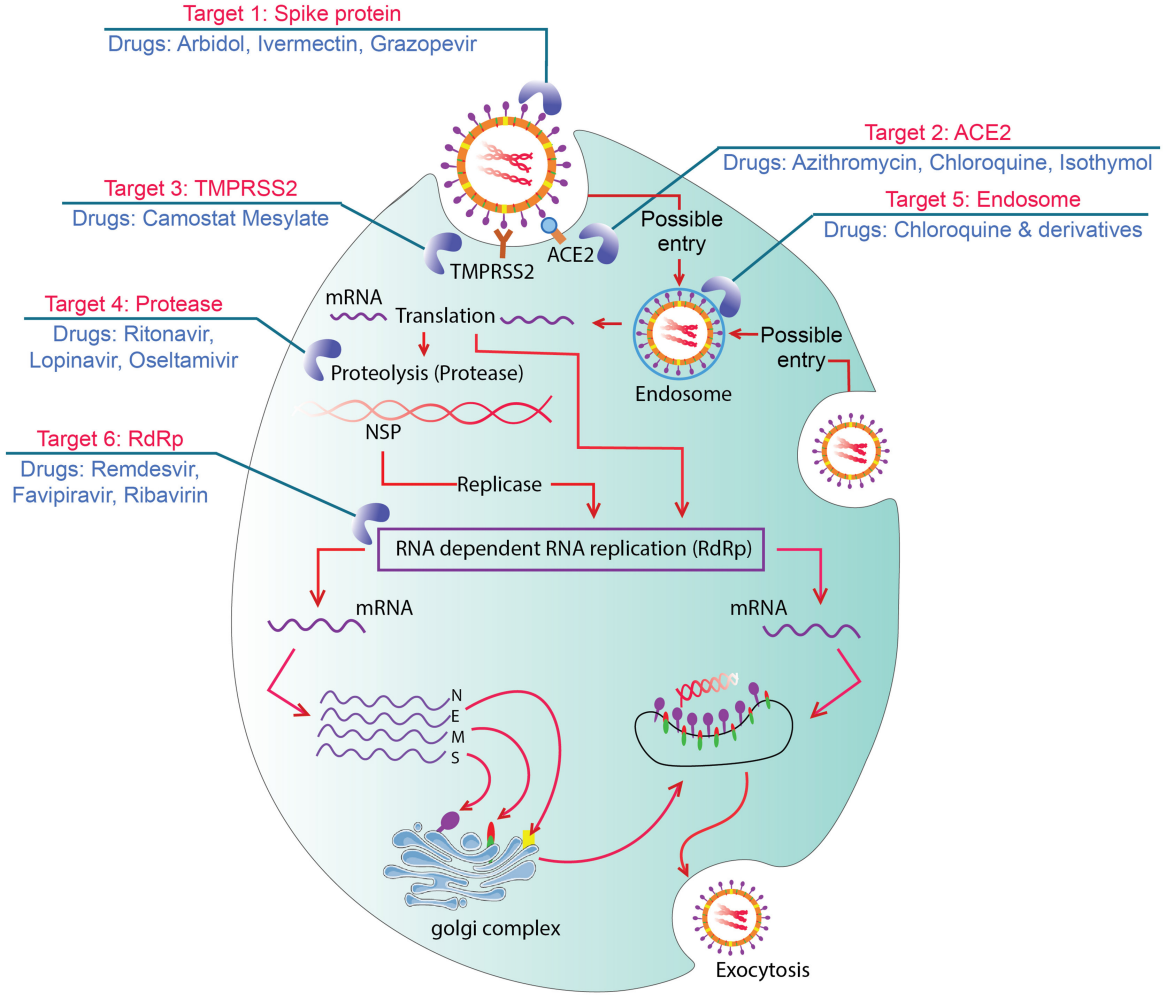
Graphic showing seven possible drug targets in viral replication process and drugs. Target 1: Spike protein blocked by arbidol and monoclonal antibodies which could bind with ACE2. Target 2: ACE2 intervened by azithromycin and chloroquine to inhibit formation of ACE2 and S protein complex. Target 3: TMPRSS2 inhibited by Camostat Mesylate to block cleavage and S protein activation. Target 4: Proteases inhibited by ritonavir, lopinavir, and/or oseltamivir that inhibit viral replication. Target 5: Endosomes targeted by chloroquine and its derivatives to increase pH and block viral RNA release from endosomes. Target 6: RdRp intervened by remdisivir, favipiravir, and/or ribavirin and cause premature termination of RNA synthesis.

After binding with angiotensin-converting enzyme (ACE)-2 by spike protein, the primary entry of SARS-CoV-2 in human cells is facilitated by protease enzyme transmembrane protease serine 2 or TMPRSS2 (Guo et al., 2020; Hoffmann et al., 2020). S1 and S2 domain of CoV-2 helps fusion (cell membranes and viral envelope) and triggers viral entry. After fusion, CoV-2 replication occurs in cell cytoplasm (Ashour et al., 2020; Mousavizadeh and Ghasemi, 2020). Spikes of CoV-2 show 10–20 times higher binding affinity with ACE-2 relative to other CoVs (Wrapp et al., 2020), and thus, ACE-2-enriched heart, lung, bronchus, nasal mucosa, kidney, ileum, stomach, and other internal organs become the primary site of CoV-2 attack leading to respiratory sickness and pneumonia (Li X. et al., 2020). The drugs and vaccines that are undergoing worldwide clinical trials have some specific targets in the host cells. ACE-2 is highly expressed in different internal and respiratory organs and is considered as a major druggable target where drugs inhibit the ACE-2 and S protein complex formation (Figure 1) (Li X. et al., 2020; Wrapp et al., 2020). Kam et al. (2009) and Shulla et al. (2011) reported viral entry and infection-facilitating human alveolar and airway protease (TMPRSS2) could be another potential target of drugs. Moreover, ongoing therapeutics trials are also targeting the interruption/inactivation of SARS-CoV-2 replication cycle, RNA release, proteases enzymes performances, inflammatory pathway activation, and development of cytokine storms in human cells (Sohag et al., 2020).

SARS-CoV-2 pathogenesis is broadly characterized as (i) entry and spread of virus, (ii) infection pathology, (iii) acute respiratory distress syndrome (ARDS), (iv) proinflammatory cytokine enhancement, and (v) dysfunction of immunity (Jin et al., 2020; Li X. et al., 2020). CoV-2 is mainly transmitted through respiratory droplets and social human contact, and primary replication occurs in the nasal cavity and pharynx with subsequent multiplication in the lower respiratory and gastrointestinal mucosa (Xiao et al., 2020). Secretion of mucus in the lungs of COVID-19 patients was not identical with previously detected SARS and MERS infections (Liu X. et al., 2020). Pathology of CoV-2 infection in lungs includes amphophilic granular cytoplasm, pulmonary edema and formation of haline membrane, mononuclear inflammatory infiltrates, increased number of lymphocytes, and enlarged pneumocytes (Mason, 2020; Xu Z. et al., 2020). These types of injuries in the lungs prevent pulmonary oxygen uptake by bronchiole, disrupt O_2_ circulation in the body, and hinder respiration collectively known as ARDS, which is fatal/lethal for the infected patients (Kaul, 2020; Mousavizadeh and Ghasemi, 2020). CoVs infections cause high virus titers and dysregulation of different proinflammatory cytokines [interleukin (IL)-1β, 8, 6; granulocyte macrophage colony stimulating factor] and chemokines [interferon-γ induced protein-10; C-C motif chemokine ligand (CCL)-2, 3, 5] termed as *cytokine storm* (Jiang et al., 2005; Cameron et al., 2008) resulting in immunopathological alteration in the lungs (Ye et al., 2020). Antiviral immune responses and over activation of T cells were determined through increased transcription of cluster of differentiation (CD)-4 and 8 (Rockx et al., 2020; Xu Z. et al., 2020). An elevated abundance of proinflammatory and cytotoxic granules is indicative of immune dysfunction in patients. It is postulated that the detection of antibody and RNA together could significantly improve the sensitivity of diagnosis for COVID-19 (Zhao et al., 2020).

**Figure 2:**
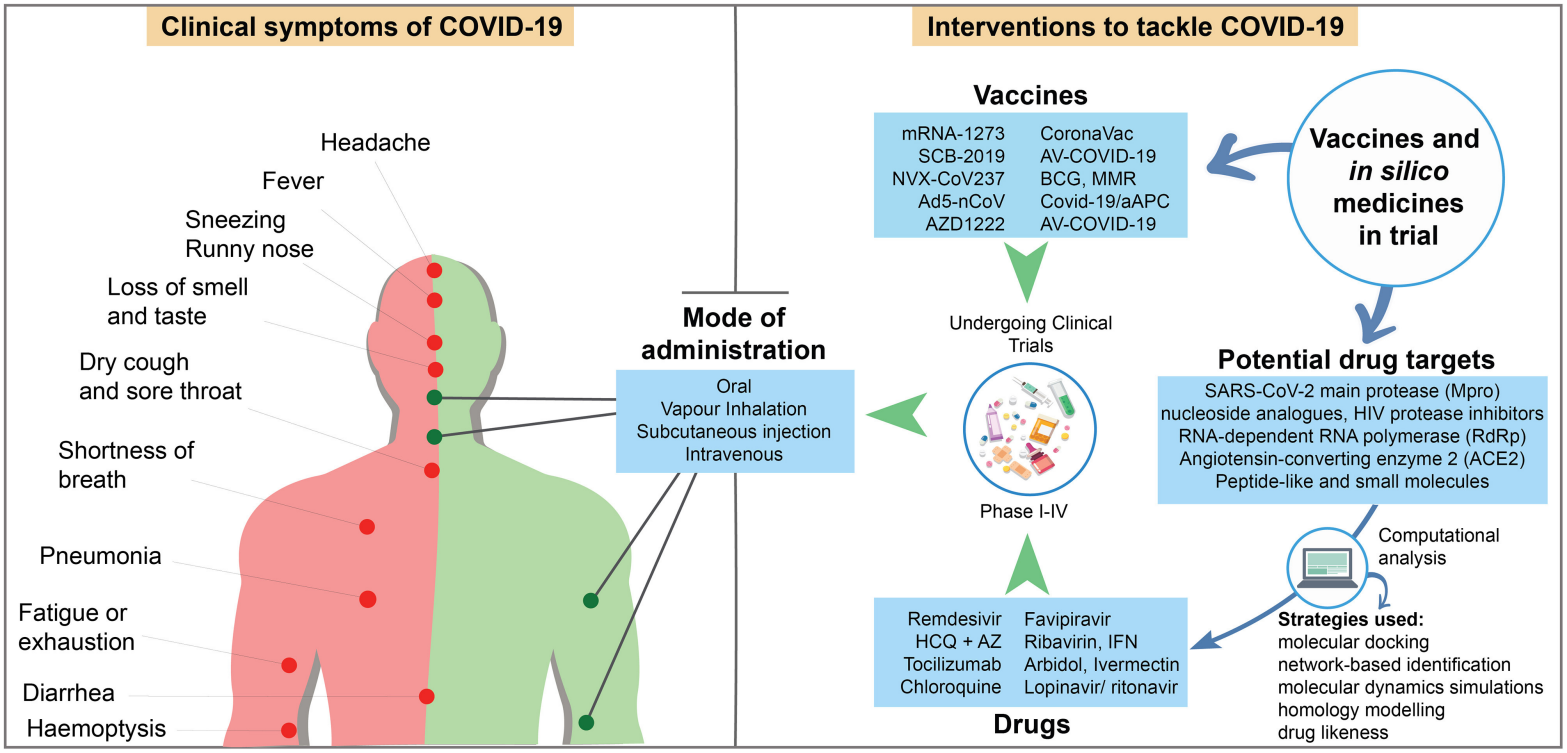
Schematic showing the symptoms of COVID-19 patients, and candidate drugs, vaccines in clinical trial, and *in silico* medicine with their mode of administration [Sources: (WHO, 2020a); CDC (Centers for Disease Control and Prevention (CDC), 2020); US NLM^1^; published reports].

The first three patients in China demonstrated severe pneumonia and two of them suffered from simple illness like fever and cough (Zhu et al., 2020). The first Cov-2 infection in the US showed basilar streaky opacities in lungs through radiography, but the pneumonia symptom was detected after 10 days (Holshue et al., 2020). Currently, UK government identified “loss of smell and taste” symptom in COVID-19 patients. Although reverse transcription quantitative PCR (RT-qPCR) is recommended for detecting SARS-CoV-2, chest CT scan may act as auxiliary method of COVID-19 diagnosis. Subsegmental consolidative area and parenchymal pulmonary ground glass opacities in lung, which are seen in the CT analysis of SARS and MERS, are also common responses to CoV-2 infection (Li X. et al., 2020). Asymptomatic human act as vectors of viral transmission and have been responsible for the rapid spreading of CoV-2. Oral and anal swabs including blood samples are typically used in CoV-2 detection. This virus can be found in oral swabs at the primary infection, anal swabs in later stage, and normal or 50% reduction in white blood cells after infection (Zhang W. et al., 2020). A study with over 400 COVID-19 patients revealed the mean incubation time of SARS-CoV-2 to be 12.5 days, which can be extended up to 24 days to induce infection symptoms (Guan et al., 2020). Among the 1,324 confirmed cases, 87.9 and 67.7% showed fever and cough, respectively (Jin et al., 2020), and 82.1% showed lymphopenia among ICU admitted patients (Yang et al., 2020). In early January 2020, the common clinical symptoms of COVID-19 among the patients of 41 hospitals in Wuhan, China included fever (98%), cough (76%), and myalgia or fatigue (45%). Among those patients, 66% had direct exposure to the Wuhan Huanan Wholesale Seafood Market, the epicenter of the COVID-19 outbreak. Symptoms including sputum production, headache, hemoptysis, and diarrhea were less frequently observed in 28, 8, 5, and 3% of patients, respectively (Huang C. et al., 2020). In addition, 96% of 138 patients (Wang D. et al., 2020) and 18% of 44 patients (Huang C. et al., 2020) demonstrated fatigue (Figure 2).

Several comorbidities like cardiovascular and cerebrovascular diseases including coagulation activation, cellular immune deficiency, myocardia, hepatic, and kidney injury with secondary bacterial infections have been observed in patients in China. To recapitulate, COVID-19 is more likely to affect older men with comorbidities who develop acute respiratory distress syndrome, resulting in worse situation and death of the patients in a short period of time (Chen N. et al., 2020).

## Vaccines in Clinical Trial

A significant development of vaccine technology has taken place in the last decade because of the invention of different candidate RNA and DNA vaccines, vectored vaccines, recombinant protein vaccines, and cell-culture-based vaccines (Amanat and Krammer, 2020). Now, it is essential to develop biologically safe and side effects free vaccines against COVID-19, for prevention and spreading of the second wave of pandemic. Because of significant genetic similarity, Liu and colleagues (Liu C. et al., 2020) mentioned that vaccines of SARS and MERS-CoVs might be effective against COVID-19. Moreover, neutralizing antibodies against S protein showed effectiveness of SARS and MERS-CoVs infection control, which has potential as an effective approach of COVID-19 treatment (Rabaan et al., 2020). However, a comparison of full-length sequence between SARS-CoV and COV-2 demonstrated most variable residues in S1 subunit of COV-2 spike (S) protein, and thus, vaccine-induced specific neutralizing antibodies effective against normal CoVs may be ineffective for controlling SARS-CoV-2 (Yu et al., 2020). Recently, convalescent plasma therapy is playing pivotal roles for CoV-2-infected critical patient recovery and prophylactic measures for the persons involved in COVID-19 treatment and patients with comorbidities (Keam et al., 2020). Plasma must be collected after 14 days after completion of COVID-19 recovery with CoV-2 neutralizing antibody (titer > 1:320). Available technology has the capacity to produce non-structural proteins, cysteine-like protease, and papain-like proteases to traverse the infected cells membrane to inhibit CoV replications, which can be considered for passive immunization of CoV-exposed and infected individuals (Dhama et al., 2020; Venkat Kumar et al., 2020).

Currently 7, 28, 5, 25, and 20 teams are working on the development of inactivated, protein subunit, virus-like particle, vector-based and DNA-RNA based vaccines, respectively, against COVID-19, although clinical trials are inadequate in response to present pandemic situation (Callaway, 2020). Previously, different types of vaccines effectively protected animals against SARS-CoV, but sometimes, live virus vaccine led to lung damage and eosinophil infiltration in mouse (Tseng et al., 2012) and liver infections in ferrets (Weingartl et al., 2004). A new vaccine typically follows R&D, clinical trials, and approval from regulatory institutions, requiring 12–18 months to be available for marketing (Verch et al., 2018).

Chen W. H. et al. (2020) broadly divided COVID-19 vaccines in trial into three groups: whole virus vaccine, subunit vaccine, and nucleic acid vaccine. Vaccine platforms against SARS-CoV-2 can be divided as RNA–DNA, recombinant protein, viral vector based, live attenuated, and inactivated vaccine (Amanat and Krammer, 2020; Dhama et al., 2020; Uddin et al., 2020). The key features of COVID-19 vaccines must have unwanted immunopotention minimization, adult health care acceptability in consideration of diabetes or hypertension, and stockpiling suitability (Chen W. H. et al., 2020).

Most vaccine-developing organizations are trying to develop CoV-2 S protein antibody in the human body by delivering S antigen through vaccine injection. mRNA-1273 is the first vaccine against COVID-19 that uses SARS-CoV-2 S protein-coded mRNA in a special type of lipid nanoparticles for injection (Hodgson, 2020). Moderna Therapeutics (Cambridge, USA) with other two organizations are jointly working for its development and clinical trial (NCT04283461 and NCT04405076) (Table 1). That vaccine has been injected first outside China to Ms. Jennifer Haller, a tech company operations manager in Seattle, USA (Cohen, 2020b). It is assumed that, after injection, host cells will uptake mRNA and produce protein in the immune system to generate responses against CoV-2 spike invasion and infection. mRNA-1273 vaccine has passed phase I and II through 105 and 600 volunteers (aged 18–54), and the recommended dose is 50 μg, but this vaccine is as yet unavailable in markets (Cohen, 2020b). Recently, vaccination of mRNA-1273 (NCT04283461) among 45 healthy adults divided in three dose groups (25, 100, and 250 μg) have induced anti-SARS-CoV-2 immune responses in all participants. In addition, no trial-limiting safety apprehensions were identified among those participants, which supports further development of the vaccine (Jackson et al., 2020). A similar type of mRNA vaccine, SCB-2019 under development by Clover Biopharmaceuticals (Changxing, China), is in phase I with a clinical trial (NCT04405908) of 150 individuals. A trimeric CoV-2 S protein is produced by mammalian cell culture, after injection might develop antibody against this virus binding with host cells to prevent infection. Its initial and day 22 recommended doses are 3 and 30 μg, respectively. The estimated completion date is March and November 2021 for SCB-2019 and mRNA-1273 vaccines^1^. Novavax (Maryland, USA) is currently researching with NVX-CoV2373 nanoparticles based vaccine at phase I in 131 individuals (NCT04368988, EudraCT2020-004123-16). At the start, Sf9 insect cells infected with baculovirus vectors to express CoV-2 S protein at cell surface resulted in immunogenic nanoparticles production. Intramuscular injection of these nanoparticles helped antigen-presenting cells to enter the local lymph nodes. In addition, currently mRNA-based vaccines from major pharmaceutical corporations like CureVac (Tubingen, Germany), Pfizer (New York, USA), and BioNTech (Mainz, Germany) are also waiting for or undertaking human clinical trials (Uddin et al., 2020). If CoV-2 S protein antibody is sufficiently strong to prevent virus engulfment by endosomes or fusion with host cells, viral protein activation and replication could be inhibited effectively.

**Table 1:** Current vaccines in clinical trials against COVID-19 [source: Biorender (Biorender, 2020) and US NLM^1^].

**Candidate vaccine (NCT ID)**	**Composition**	**Mode of action**	**Dose**	**Clinical trial (volunteers)**	**Country and company/institute/organization**
mRNA-1273 (NCT04283461, NCT04405076)	SARS-CoV-2 S protein encoded mRNA in lipid nanoparticle	Immune response against Cov-2 S protein	50 μg	Phase I (105) Phase II (600)	Moderna, NIAID, Biomedical Advanced Research and Development Authority
SCB-2019 (NCT04405908)	CoV-2 S proteins trimer produced by mammalian cell culture	Antibodies against CoV-2 to prevent binding and infection	3 and 30 μg at days 1 and 22, respectively	Phase I (150)	Clover Biopharmaceuticals
NVX-CoV2373 (NCT04368988, EudraCT2020-004123-16)	Insect cells infections to express CoV-2 S protein.	Antigen presentation in the local lymph nodes	25 μg at days 1 and 22	Phase I (131)	Novavax
CoronaVac (NCT04352608, NCT04383574)	Inactivated SARS-CoV-2	Diverse immune response against numerous viral antigens	300 SU/ml antigen at days 1 and 29	Phase I (216) Phase II (950)	Sinovac Biotech Co.
Ad5-nCoV (NCT04313127, NCT04341389, NCT04398147, ChiCTR2000031781, ChiCTR2000030906)	Replication inactive adenovirus	Antibodies production against CoV-2 S protein.	1 ml injection in the deltoid muscle at day 1 (1 × 10^11^ vp)	Phase I (108) Phase I/II (696) Phase II (508)	CanSino Biologics, Institute of Biotechnology, Academy of Military Medical Sciences, China
ChAdOx1 nCoV-19 (NCT04324606, NCT04400838, EudraCT 2020-001072-15, EudraCT 2020-001228-32)	Attenuated adenovirus	Endogenous antibodies protection against SARS-CoV-2	A single dose of 5 × 10^10^ vp	Phase I/II (1,090) Phase II/III (10,260)	Consortium of the Jenner Institute, Oxford Biomedical Research Center, University of Oxford
Bacille Calmette-Guérin (NCT04387409 and another 13)	Live attenuated *Mycobacterium bovis*	Immune responses against *M. tuberculosis* infection	2–8 × 10^5^ CFU injection in 0.1 ml suspension	Phase III (18,798) Phase IV (2,800)	University Medical Center Utrecht, Radboud University and other organizations
Measles, mumps, and rubella (MMR) (NCT04357028)	Live-attenuated measles, mumps, and rubella virus	Cross reaction with SARS-CoV-2	0.5 ml	Phase III (200)	Cairo University Hospital Cairo, Egypt
INO-4800 (NCT04336410)	DNA plasmid that encodes S protein antigens of CoV-2	T cells, B cells, and encoded proteins production	1.0 mg ID injection at day 0 and week 4	Phase I (40)	Inovio Pharmaceuticals
AV-COVID-19 (NCT04386252)	DC and GM-CSF from blood monocytes	Non-mentioned	1 × antigen with/without 500 μg GM-CSF	Phase I/II (180)	Aivita Biomedical, Inc.
Covid-19/aAPC (NCT04299724)	Lentivirus modified DC, immune modulatory genes, and CoV-2 minigenes	Priming T lymphocytes against CoV-2	Three subcutaneous injections 5 × 10^6^ cells	Phase I (100)	Shenzhen Geno-immune Medical Institute Shenzhen, Guangdong, China
LV-SMENP-DC (NCT04276896)	DC modification with lentivirus vectors to express SMENP	Priming T lymphocytes against CoV-2	5 × 10 ^6^ cells (subcutaneous) and antigen specific 1 × 10^8^ CTLs (IV infusion)	Phase II (100)	Shenzhen Geno-immune Medical Institute Shenzhen, Guangdong, China

*S, Spike; SU, subunit; vp, vaccine particle; ID, intradermal; DC, dendritic cell; GM-CSF, granulocyte-macrophage colony-stimulating factor; SMENP, Shenzhen Minigene Engineered-NP; IV, intravenous; CTLs, cytotoxic T lymphocyte*.

SARS-CoV-2 propagation through cell culture and then inactivated by propiolactone has turned into a vaccine, CoronaVac, developed by Sinovac Biotech Co. (Beijing, China). This vaccine may produce diverse immune responses against CoV-2 and is not harmful in human trials (NCT04352608, NCT04383574) because of the use of inactivated virus. It is in phase II trial with a recommended dose 300 SU/ml day at day 1 and after 4 weeks. The final result of this trial could be in hand by July 2020 (Table 1). Similarly, inactivated adenovirus vaccine Ad5-nCoV (CanSino Biologics, China) is now in phase II by assuming the possibilities of antibodies development to prevent CoV-2 entrance through spikes in human cells. Its phase I clinical trial (NCT04313127, ChiCTR2000031781, ChiCTR2000030906) involved 108 volunteers (aged 18–60 years) in Wuhan, China and is expected to be completed in December 2022 (Thanh Le et al., 2020). Another attenuated adenovirus vaccine, ChAdOx1 nCoV-19, is under phase II/III developed by University of Oxford, UK. Its composition and mechanisms of action are identical to other S-protein-based vaccines like mRNA-1273 and SCB-2019. ChAdOx1 nCoV-19 recommended one singular dose at 5 × 10^10^ vaccine particle (vp), but SCB-2019 described different doses at different times (Table 1). Moreover, the Lancet journal published preliminary findings of ChAdOx1 nCoV-19 (NCT04324606, EudraCT 2020-001072-15, EudraCT 2020-001228-32) on safety, reactogenicity, and cellular and humoral immune responses on July 20, 2020. The results revealed a satisfactory safety profile, and homologous boosting increased antibody responses. ChAdOx1 nCoV-19 resulted in the induction of both humoral and cellular immune responses against SARS-CoV-2, with increased responses after a second dose supports largescale evaluation of this candidate vaccine in an ongoing phase III program. Besides, further clinical studies with older adults were recommended by the authors (Folegatti et al., 2020).

Bacille Calmette-Guérin (BCG) and measles, mumps, and rubella (MMR) are vaccines of live attenuated *Mycobacterium bovis* and MMR-specific viruses. Previously, it was ensured that BCG and MMR not only prevent their respective infections but also reduced severity and morbidity through cross-reaction of other respiratory diseases/infections. Already, 14 human trials of BCG by different research organizations are completed and continuing in phase IV. MMR is in phase III with clinical trial (NCT04357028) of 200 people at a dose of 0.5 ml subcutaneous injection. Both vaccines are not against CoV-2 but may reduce the respiratory sickness induced among COVID-19 patients.

The Bill and Melinda Gates Foundation sponsored a DNA vaccine, INO-4800, developed by Inovio Pharmaceuticals (Plymouth Meeting, USA). Cellectra Technology (electric impulse) is used to deliver this S protein encoded plasmid antigen intracellularly by creating a small pore for easier uptake. Treated subjects may create antibodies against CoV-2 based on codes in DNA plasmid, and it is now in phase I clinical trial (NCT04386252) at a dose of 1.0 mg intradermal (ID) injection at 0 and 4 weeks. To produce AV-COVID-19 vaccine, initially, blood monocytes are differentiated into dendritic cells (DC) by IL-4 and granulocyte-macrophage colony-stimulating factor (GM-CSF) after incubation with CoV-2 antigen. Aivita Biomedical (California, USA) developed AV-COVID-19, which is undergoing phase I/II clinical trial (NCT04386252) with 180 volunteers and is estimated for completion in April 2021^1^.

Shenzhen Geno-immune Medical Institute, China developed Covid-19/artificial antigen-presenting cells (aAPCs) and LV-SMENP-DC vaccines by the lentivirus-modified DC, immune modulatory genes, and CoV-2 viral minigenes (SMENP). The mode of actions of both vaccines are priming of T lymphocytes against CoV-2. In the case of Covid-19/aAPC, the subject will get three subcutaneous injections (5 × 10^6^ cell), and this treatment is now in phase I trial with 100 volunteers (NCT04299724). Initially, human trial (NCT04276896) of LV-SMENP-DC used a single dose of Covid-19/aAPC injection (Table 1) followed by 1 × 10^8^ cytotoxic T lymphocytes (CTLs) intravenous infusion. Patients are followed up weekly for 1 month, monthly for 3 months, and then every 3 months after infusion, until the end of trial.

## Drugs in Clinical Trial

Remdesivir, a nucleotide analog prodrug that is intracellularly metabolized to adenosine triphosphate, could inhibit SARS-CoV-2 and MERS-CoV and non-clinically demonstrated therapeutic efficacy against viruses (Grein et al., 2020). Two clinical trials have referred to the use of remdesivir in COVID-19 patients (Rosa and Santos, 2020). The clinical efficacy and safety of remdesivir or placebo among COVID-19-positive adult patients were assessed in 60 trial sites and 13 subsites worldwide including the UK and US. A double-blind, randomized placebo-controlled intravenous administration of remdesivir at a dose of 200 mg on day 1 followed by 100 mg of placebo for additional 9 days was assigned at a 1:1 ratio to receive either remdesivir or placebo. During the trial, all patients received standard supportive care from hospitals from day 1–29. In a 10-days course, remdesivir was suggested to be superior to placebo in terms of recovery. Besides this, no associated deaths were reported in the trials except some serious adverse events in 21.1 and 27% patients in remdesivir and placebo group, respectively. However, early unblinding of the results were recommended by the data and safety monitoring board (Beigel et al., 2020). In addition, patients having <94% oxygen saturation or receiving oxygen support were provided remdesivir at the aforementioned dose, and 68% of oxygen-support class experienced an improvement, while only 57% of mechanical ventilation subjects improved, and the death rate was 13% (Grein et al., 2020). Similar findings were reported from a 40-year-old patient admitted to the hospital with 2-days history of dry cough, shortness of breath, and subjective fever supplemented with 100 mg intravenous remdesivir at every 24 h for 9 days after 13th days of illness. The patient started to progress after 48 h and became stable at room air at 17th day of illness leading to discharge from the hospital (Hillaker et al., 2020). The combined effect of remdesivir and emetine also confirmed antiviral activity to inhibit SARS-CoV-2 *in vitro* (Choy et al., 2020).

An experimental combination of treatments has been used in response to an outbreak in the US White House in response to President Trump's COVID-19 infection (Cohen, 2020a). Remdesivir was administered intravenously under emergency approval. An experimental combination of monoclonal antibodies produced by Regeneron was also used, the US Food and Drug Administration terms for *Expanded Access*, to allow treatment with experimental drugs. These antibodies are believed to interfere with viral binding to receptors in human cells. Added to this, a combination of monoclonal antibodies that act upon different epitopes on viral surface could be analyzed. A variety of monoclonal antibodies that inhibited SARS-CoV (e.g., 80R, CR3014, CR3022, m396, 4D4, etc.) and MERS-CoV (e.g., MERS-4, MERS-27, m336, G4, D12 etc.) could be tested against SARS-CoV-2. However, producing monoclonal antibodies in large scale is labor intensive, expensive, and time consuming (Shanmugaraj et al., 2020). Dexamethasone was also provided to subdue pathological immune responses sometimes associated with COVID-19. The results of these treatments appear promising but have thus far been kept confidential; the online SCIENCE article is likely to be updated.

Hydroxychloroquine (HCQ) and chloroquine (CQ) are antimalarial drugs having antiviral action against HIV by inhibiting entry into host cells (Rosa and Santos, 2020). In addition, they can alter post-translation of newly synthesized protein by inhibiting glycosylation (Rolain et al., 2007). Gautret and colleagues evaluated the effect of HCQ on respiratory viral loads in over 12 years old COVID-19 patients in the hospital of Marseille, South France. All patients were given 200 mg of HCQ orally thrice daily during the 10-days trial and six of them received azithromycin (AZ; control group) for preventing bacterial infection. The combined treatment of HCQ + AZ, HCQ, and control resulted in 100, 57.1, and 12.1% cure, respectively. Therefore, HCQ is promising to reduce viral load in COVID-19 patients that is further strengthened by addition of AZ (Gautret et al., 2020). Another trial involving 1,061 patients lasted at least 3 days and a maximum of 10 days with HCQ + AZ and resulted in 92% of improved clinical condition with reduced viral load within 10 days, except for 4% poor clinical condition and 1% death (Million et al., 2020). A recent New England Journal of Medicine report (3 June 2020) revealed ineffectiveness of HCQ to prevent illness related to or confirmed COVID-19 cases. Here, HCQ was used in a double-blind, placebo-controlled trial as post-exposure prophylaxis among 821 asymptomatic patients in the US and Canada within 4 days after exposure. Subsequent illness related to COVID-19 or laboratory-confirmed incidences were reported after 14 days of treatment (Boulware et al., 2020).

CQ was found to be a potential antiviral agent in 2006 (Savarino et al., 2006) and active against CoV-2 infection at lower micromolar concentrations (Wang M. et al., 2020). Patients affected by COVID-19 were discharged from the hospital more quickly after receiving 500 mg of CQ twice daily with 400 mg/100 mg/capsule of lopinavir/ritonavir. In addition, the clearance of lung and CoV-2 negative result even after 2 days of treatment was notable. On the other hand, some responses were reported, including vomiting (most common), abdominal pain, diarrhea, nausea, rash, cough, and shortness of breath. In general, CQ was well-tolerated by the patients (Dong L. et al., 2020; Huang M. et al., 2020), and it can modulate immunity in the whole body including the lungs, which demonstrated enhanced antiviral activity (Wang M. et al., 2020).

Favipiravir is another nucleoside analog that has been used previously against SARS- and MERS-CoVs, although the efficacy is debatable (Cai et al., 2020). In recent times, favipiravir inhibited SARS-CoV-2 in Vero E6 cells, where its half-maximal effective concentration (EC_50_) was 67 μmol L^−1^ (Wang M. et al., 2020) and was found to prevent mice from infection with the Ebola virus (Oestereich et al., 2014). Recently, 120 patients from Wuhan, China were assigned to receive favipiravir for 10 days in a controlled, randomized, and open-label trial. Consequently, patients experienced relief from pyrexia and cough in shorter latencies leading to early clinical recovery (Chen C. et al., 2020). In another trial, favipiravir was assessed after 14 days of administration, which resulted in viral clearance in shorter time than the control group along with notable improvement in chest computed tomography (CT). In the adverse event, favipiravir-treated group suffered less than the control group, 11.43 and 55.56%, respectively (Cai et al., 2020). Furthermore, a phase III randomized, double-blind trial of favipiravir is ongoing with 50 participants in Bangladesh, and the trial is expected to be completed in July, 2020 (clinicaltrials.gov). Participants will be assessed by primary and secondary outcome measures, which include negative results of viral presence, change in lung condition, and clinical recovery.

Interferon (IFN) reportedly inhibits SARS CoVs, although it is used to treat hepatitis (Stockman et al., 2006). The specific method for administration of IFN-α is vapor inhalation at a dose of 5 million international units (MIU) with 2 ml of sterile water for injection with a frequency twice daily (Dong L. et al., 2020). A combination of IFN-β1b with ribavirin, lopinavir, and ritonavir through subcutaneous injection and nasogastric tube have brought about better clinical conditions and viral clearance (Table 2). During a 14-days trial, a combination of ribavirin (400 mg) at every 12 h, lopinavir (400 mg), and ritonavir (100 mg) every 12 h, three doses (8 MIU) of IFN-β1b on alternate days (combination group), or only the above-mentioned dose of lopinavir and ritonavir (control group) were supplemented. Overall, the combination treatment eased mild to moderate COVID-19 symptoms (Figure 2) with no serious adverse events and reduced the time of viral shedding and the duration of hospital stay (Hung et al., 2020).

**Table 2:** Current drugs in clinical trials against COVID-19.

**Drug**	**Dosage**	**Mode of administration**	**Trial phase**	**Result/outcome**	**References**
Remdesivir or placebo	200 mg on day 1 followed by 100 mg daily for 9 more days	Intravenous	3	Shortened recovery time No associated mortality	Beigel et al., 2020
Remdesivir	200 mg on day 1 followed by 100 mg daily for 9 more days	Intravenous	3	Improved breathing and clinical conditions	Grein et al., 2020
Remdesivir	100 mg at every 24 h for 9 days	Intravenous	3	Improved breathing Became stable at room air	Hillaker et al., 2020
Hydroxychloroquine (HCQ) sulfate + Azithromycin (AZ)	200 mg HCQ thrice daily, with AZ (500 mg daily on day 1, 250 mg on days 2–5), 10 days	Oral	3	Reduce viral carriage Effect reinforced by addition of AZ	Gautret et al., 2020
HCQ + AZ	200 mg HCQ for 10 days with 5 days of AZ (500 mg daily on day 1, 250 mg on day 2–5), 10 days	Oral	3	Low proportion of adverse events of patients with mild symptoms	Million et al., 2020
Chloroquine (CQ) phosphate + Lopinavir/ritonavir	500 mg of CQ, 400 mg/100 mg/capsule of lopinavir/ritonavir, twice daily for 10 days	Oral	4	Quick discharge from hospital Few adverse events	Dong L. et al., 2020
CQ + Lopinavir/ritonavir	500 mg CQ with 400/100 mg of lopinavir/ ritonavir, twice daily, 10 days	Oral	4	Achieved lung clearance Became SARS-CoV-2 negative after 2 days	Huang M. et al., 2020
Favipiravir	1,600 mg twice in day 1 followed by 600 mg twice daily for days 2–10	Oral	2	Relieved pyrexia and cough Raised uric acid in serum	Chen C. et al., 2020
Favipiravir	1,600 mg twice in day 1 followed by 600 mg twice daily for days 2–14	Oral	2	Shortened viral clearance duration	Cai et al., 2020
IFN-α	5 million units (U) + 2 ml sterile water for injection, twice daily, 10 days	Vapor inhalation	N/A	Not mentioned	Dong L. et al., 2020
Ribavirin + IFN-β1b or Lopinavir/ritonavir	400 mg ribavirin at every 12 h, 8 million IU of IFN-β1b on alternate days, or 400 mg lopinavir and 100 mg ritonavir at every 12 h, 14 days	Subcutaneous injection, via nasogastric tube	2	Better virological and clinical condition No serious adverse events	Hung et al., 2020
Arbidol	200 mg, thrice daily, 10 days	Oral	4	Not mentioned	Dong L. et al., 2020
Arbidol (Umifenovir)	200 mg, thrice daily, 10 days	Oral	4	Relieved pyrexia and cough	Chen C. et al., 2020
Tocilizumab	400 mg diluted with 100 ml 0.9% normal saline, twice daily, 10 days	Intravenous	3	Body temperature returned to normal Relieved clinical symptoms	Xu X. et al., 2020
Nafamostat	200 mg; 24 h continuously with acetaminophen	–	-	CRP level decreased SARS-CoV-2 negative	Jang and Rhee, 2020

Arbidol, also known as umifenovir, is another influenza virus inhibitor patented for SARS treatment with no adverse effects (Wang M. et al., 2020) and is undergoing four clinical trials with basic treatment, oseltamivir, rotinavir–liponavir, and carrimycin (Rosa and Santos, 2020). Among them, oseltamivir is an approved drug for inhibiting influenza A and B by blocking viral entry and reducing their spreading in respiratory tract (Uyeki, 2018). Besides these, a clinical trial with 124 patients in Wuhan, China reported a 34.1% discharge of the patients, while 61.6% remain hospitalized. Major complications during hospital stay was ARDS, arrhythmia, and shock (Wang M. et al., 2020). Additionally, favipiravir revealed significant improvement of patients over arbidol in another trial (Chen C. et al., 2020). Tocilizumab, an IL-6 inhibitor, administered to patients from 5 to 14 February 2020, resulted in dramatic normalization of body temperature within 2 days of treatment. In addition, the clinical symptoms and peripheral oxygen saturation improved substantially after 5 days of treatment (Xu X. et al., 2020).

According to US NLM, most of the aforementioned drugs, viz. remdesivir, HCQ, AZ, and tocilizumab, are in phase III of clinical trials. Unfortunately, two of the remdesivir trials were postponed or terminated due to no eligible patients for the clinical study. On 2 June 2020, CQ and arbidol are recruiting in phase IV of the trial, although some (ribavirin, favipiravir) are still in phase II recruitment (Table 2).

The majority of the deaths associated with SARS-CoV-2 is convoluted with coagulopathy and disseminated intravascular coagulation (DIC) according to recently published reports. Besides, patients with severe COVID-19 are attacked by sepsis 3 (Singer et al., 2016) and venous thromboembolism (VTE) due to severe virus infection, respiratory dysfunction, and long-term bed rest including hormone treatment, respectively. Despite the continuing need to validate the efficacy, anticoagulants (e.g., heparin) have been recommended by experts (Cai et al., 2020; Huang M. et al., 2020). As for instance, among 449 patients in Tongji Hospital Wuhan, 99 patients received heparin as anticoagulant, where 94 patients received low molecular weight heparin (LMWH) with a dose of 40–60 mg enoxaparin/day and 10,000–15,000 U/day of unfractioned heparin, having no specific inclusion or exclusion criteria pointed out until now, with mortality of heparin users lower than that of the non-heparin users. Therefore, the scientists group found an association of low molecular weight heparin with severe COVID-19 patients' prognosis (Oestereich et al., 2014; Huang M. et al., 2020). To conclude, besides the current treatment of severe and critical COVID-19 patients, early initiation of IVIg and LMWH anticoagulant therapies are prescribed to control the number of deaths (Stockman et al., 2006; Huang M. et al., 2020).

According to a recently published article, an anticoagulant, namely, nafamostat, having potential anti-inflammatory and antiviral activities against COVID-19, has been tested on three elderly patients with acetaminophen. A dose of 200 mg of nafamostat was administered continuously for 24 h. Consequently, the C-reactive protein (CRP) level of the patients was found to be decreased from 2.61 to 1.32 mg/dl in patient 1, and similar results were also shown by the other two patients. On top, all the patients initially required oxygen; after administering nafamostat, however, they maintained 98% oxygen saturation without supplementation (Jang and Rhee, 2020).

## *In silico* Medicines

*In silico* approaches have gained more acceptance for their diverse applicability in molecular biology (Ekins et al., 2007; Yuriev et al., 2011; Papadatos and Brown, 2013). It is a rapidly growing area that is primarily used to identify, analyze, and integrate chemical, biological, and medical data using different software or online database and repositories (Husmeier et al., 2006; Kuhn et al., 2008). *In silico* drug repurposing strategy is an efficient method of suggesting from already approved drugs against lethal pathogens by using machine learning computational techniques and bioinformatics tools (Chang et al., 2007; Veljkovic et al., 2015; Shah et al., 2020). Over the years, chemical and biological data have been generated at an accelerated pace, marking the arrival into the “big data” era (Costa, 2014; March-Vila et al., 2017). This leads the scientific community to acquire new opportunities to link drugs to diseases, although this relationship is indirect and depends on complex mechanisms of action. Therefore, an improved understanding of the associations between drugs and their targets, and between targets and diseases, is essential for *in silico* drug discovery (Trivedi et al., 2020). During health complications induced by COVID-19, the entire world is eagerly anticipating treatment options against SASR-CoV-2, but the outcome remains uncertain. In this situation, the scientific community is trying to reuse the approved drug against COVID-19 by high throughput screening of different approved drug molecules against different probable drug targets of SARS-CoV-2 (Table 3). A huge number of *in silico* studies have already done in search of effective therapeutics against COVID-19, and a moderate number of drugs and suggested compounds are being tested under laboratory conditions or even in clinical trials with COVID-19 patients (Table 3).

**Table 3:** Suggested *in silico* medicines against COVID-19.

**Targets for SARS-CoV-2**	**Suggested drug**	***In silico* method**	**FDA approval**	**Clinical trial**	**References**
Main Protease	3-Phenyllactic Acid, Chrysin, Caffeic Acid, Galangin, Lumichrome	MD	No	No	Hashem, 2020
	Caffeic Acid Phenylethyl Ester (Cape)	MD	No	Yes	Hashem, 2020
	Abt450, Asunaprevir, Azidothimidine, Cgp42112A, Faldaprevir, Galidesivir, Marboran/Methisazone, Mericitabine, Nsc306711 (Ferristatin Ii), Ravidasvir, Simeprevir, Uprifosbuvir, Vedroprevir	MD	No	No	Shah et al., 2020
	Baricitinib, Daclatasvir, Sofosbuvir	MD	Yes	Yes	Shah et al., 2020
	Danoprevir	MD	No	Yes	Shah et al., 2020
	Amprenavir, Delavirdine, Didanosine, Efavirenz, Elbasvir, Elvitegravir, Entecavir, Famciclovir, Grazoprevir	MD	Yes	No	Shah et al., 2020
	Aloe-Emodin, Withanolide D	MD	No	No	Chandel et al., 2020
	Enoxacin, Withaferin	MD	Yes	No	Chandel et al., 2020
	Rhein	MD	Yes	Yes	Chandel et al., 2020
	Artemisinin, Quinine	MD, PASS	Yes	Yes	Srivastava et al., 2020
	Mepacrine	MD, PASS	Yes	No	Srivastava et al., 2020
	Phomarin, Proguanil	MD PASS	No	No	Srivastava et al., 2020
	Betulinic Acid, Coumaroyltyramine, Cryptotanshinone, Desmethoxyreserpine, Dihomo-C-Linolenic Acid, Kaempferol, Moupinamide, N-Cis-Feruloyltyramine, Sugiol, Tanshinone Iia	MD	No	No	Zhang D. et al., 2020
	Lignan, Quercetin	MD	No	Yes	Zhang D. et al., 2020
	Birinapant, Leupeptin Hemisulphate, Lypression, Pepstatin A	MDS	No	No	Mittal et al., 2020
	Octreotide	MDS	Yes	Yes	Mittal et al., 2020
	Dpnh (Nadh), Flavin Adenine Dinucleotide (Fad) Adeflavin	MD, HM	No	No	Hall and Ji, 2020
	Bortezomib, Cangrelor, Carfilzomib	MD, HM	Yes	No	Hall and Ji, 2020
	Camphor, Melatonin	NBI	No	Yes	Zhou et al., 2020
	Carvedilol, Dactinomycin, Irbesartan, Mercaptopurine, Paroxetine, Oxymetholone, Toremifene	NBI	Yes	No	Zhou et al., 2020
	Colchicine, Eplerenone, Sirolimus	NBI	Yes	Yes	Zhou et al., 2020
	Emodin, Equilin, Mesalazine, Quinacrine	NBI	No	No	Zhou et al., 2020
	Chloroquine	MD, PASS	Yes	Yes	Arya et al., 2020; Srivastava et al., 2020
	Cobicistat	MDS	Yes	Yes	Pant et al., 2020
	Cyanidin, Daidzein, Genistein, Phycocyanobilin,	MD	No	No	Pendyala and Patras, 2020
	Riboflavin	MD	Yes	No	Pendyala and Patras, 2020
	Darunavir	MDS	Yes	Yes	Mohamed et al., 2020; Ortega et al., 2020; Pant et al., 2020
	Elbasvir	MD	Yes	No	Cavasotto and Filippo, 2020
	Fluvastatin	MD	Yes	No	Biembengut and de Arruda Campos Brasil de Souza, 2020; Reiner et al., 2020
	Hydroxychloroquine	MD, PASS	Yes	Yes	Barros et al., 2020; Mamidala et al., 2020; Srivastava et al., 2020
	Indinavir	MD	Yes	Yes	Biembengut and de Arruda Campos Brasil de Souza, 2020; Hall and Ji, 2020; Mamidala et al., 2020; Mohamed et al., 2020; Shah et al., 2020
	Lopinavir	MD	Yes	Yes	Barros et al., 2020; Biembengut and de Arruda Campos Brasil de Souza, 2020; Chen Y. W. et al., 2020; Kumar and Singh, 2020; Mohamed et al., 2020; Mothay and Ramesh, 2020; Ortega et al., 2020; Pant et al., 2020; Shah et al., 2020
	Lovastatin	MD	Yes	Yes	Enayatkhani et al., 2020; Reiner et al., 2020
	Lumichrome	MD	No	No	Hashem, 2020
	Amprenavir	MD	Yes	No	Ortega et al., 2020
	Zinc000000702323, Zinc000012481889, Zinc000015988935, Zinc000103558522, Talampicillin	MDS	No	No	Elmezayen et al., 2020
	Lurasidone, Rubitecan	MDS	Yes	No	Elmezayen et al., 2020
	Tmprss2	MDS	Yes	Yes	Elmezayen et al., 2020
	Nelfinavir	MD	Yes	No	Biembengut and de Arruda Campos Brasil de Souza, 2020; Chandel et al., 2020; Kumar and Singh, 2020; Mittal et al., 2020; Mohamed et al., 2020; Mothay and Ramesh, 2020
	Oseltamivir	MD	Yes	Yes	Mamidala et al., 2020; Shah et al., 2020
	Pitavastatin	MD	Yes	No	Reiner et al., 2020
	Raltegravir	MD	Yes	No	Kumar and Singh, 2020; Sencanski et al., 2020; Shah et al., 2020
	Remdesivir	MD	Yes	Yes	Hall and Ji, 2020; Mothay and Ramesh, 2020; Shah et al., 2020
	Rifampicin	MD	No	Yes	Pathak et al., 2020
	Ritonavir	MDS	Yes	Yes	Barros et al., 2020; Chen Y. W. et al., 2020; Kumar and Singh, 2020; Mothay and Ramesh, 2020; Ortega et al., 2020; Pant et al., 2020; Shah et al., 2020
	Rosuvastatin	MD	Yes	Yes	Biembengut and de Arruda Campos Brasil de Souza, 2020; Reiner et al., 2020
	Saquinavir	MD	Yes	No	Barros et al., 2020; Biembengut and de Arruda Campos Brasil de Souza, 2020; Hall and Ji, 2020; Ortega et al., 2020; Shah et al., 2020
	Telaprevir	MD	Yes	No	Mohamed et al., 2020; Shah et al., 2020
	Tenofovir	MD	Yes	Yes	Kumar and Singh, 2020; Shah et al., 2020
	Zanamivir	MD	Yes	No	Hall and Ji, 2020; Shah et al., 2020
Spike Glycoprotein	Alafenamide, Aprotinin, Artesunate, Bedaquiline, Cefpiramide, Desmopressin, Erythromycin, Fostamatinib, Hydroxychloroquine	MD	Yes	No	Bank et al., 2020
	Amyrin, Loniflavone, Phillyrin, Proanthocyanidin, Procyanidin, Punicalagin, Sericoside, Strictinin, Tirucallina	MD, SML	No	No	Kadioglu et al., 2020
	Everolimus	MD, SML	Yes	Yes	Kadioglu et al., 2020
	Nystatin, Paritaprevir, Simeprevir	MD, SML	Yes	No	Kadioglu et al., 2020
	Rutin	MD, SML	No	Yes	Kadioglu et al., 2020
	Apigenin, Curcumin, Fisetin, Genistein, Isorhamnetin, Kamferol, Luteolin, Pterostilbene, Quercetin, Resveratrol	MD	No	No	Rane et al., 2020
	Cangrelor	MD, HM	Yes	No	Hall and Ji, 2020
	Coenzyme A, Dpnh (Nadh), Flavin Adenine Dinucleotide (Fad) Adeflavin, Iomeprol	MD, HM	No	No	Hall and Ji, 2020
	Dihydrotanshinonei	ADME, MD	No	No	Zhang D. et al., 2020
	Grazoprevir	MD, SML	Yes	No	Ibrahim et al., 2020; Kadioglu et al., 2020; Mohamed et al., 2020; Shah et al., 2020
	Ivermectin	MD, SML	Yes	Yes	Ibrahim et al., 2020; Kadioglu et al., 2020
	Teniposide	MD, SML	Yes	No	Chen Y. W. et al., 2020; Kadioglu et al., 2020
	Velpatasvir, Ledipasvir	MD, SML	Yes	Yes	Chen Y. W. et al., 2020; Kadioglu et al., 2020
	Rifabutin	MD, SML	Yes	Yes	Bank et al., 2020; Kadioglu et al., 2020
	Saikosaponins U, Saikosaponins V	MD	No	No	Sinha et al., 2020
Nucleocapsid protein	Conivaptan, Ergotamine, Rifabutin	MD, SML	Yes	Yes	Kadioglu et al., 2020
	Dihydroergotamine, Eribulin, Natamycin, Nystatin, Rifapentine, Valrubicin	MD, SML	Yes	No	Kadioglu et al., 2020
	Euphol, Forsythiaside, Ilexsaponinb2, Ilexsaponinb3, Procyanidin, Punicalagin, Sericoside, Strictinin, Tirucallina,	MD, SML	No	No	Kadioglu et al., 2020
	Cvl218	MD, HM	No	No	Ge et al., 2020
	Nelfinavir (Viracept)	MD	Yes	No	Musarrat et al., 2020
	Olaparib	MD, HM	No	No	Ge et al., 2020
	Venetoclax	MD, SML	Yes	No	Chen Y. W. et al., 2020; Kadioglu et al., 2020
	Zinc0000146942, Zinc00003118440	MDS	No	No	Sarma et al., 2020
RNA-dependent RNA Polymerase	Cefuroxime	MD, DS	Yes	No	Elfiky, 2020
	Galidesivir, Idx-184, Setrobuvir, Yak	MD, DS	No	No	Elfiky, 2020
	Hydroxychloroquine, Sofosbuvir, Tenofovir	MD, DS	Yes	Yes	Elfiky, 2020
	Favipiravir	MD, DS	No	Yes	Elfiky, 2020; Reiner et al., 2020
	Remdesivir	MD, DS	Yes	Yes	Elfiky, 2020; Mohamed et al., 2020
	Ribavirin	MD, DS	Yes	Yes	Elfiky, 2020; Mohamed et al., 2020
	Silybin (Silybum marianum), Withaferin	MD	No	No	Pandit and Latha, 2020
Envelope Protein	Belachinal, Macaflavanone E, Vibsanol B	MD	No	No	Gupta et al., 2020
2′-o-ribose-methyltransferase	3,4,-Dicaffeoylquinic Acid, 3,5-Dicaffeoylquinic Acid, 4,5, Dicaffeylquinic Acid, Procyanidin, Punicalagin, Strictinin, Tirucallina, Tingeninb, Loniflavone	MD, SML	No	No	Kadioglu et al., 2020
	Dihydroergotamine, Paritaprevir, Venetoclax, Tenoposide	MD, SML	Yes	No	Kadioglu et al., 2020
	Ergotamine, Ivermectin, Nilotinib, Posaconazole, Telithromycin	MD, SML	Yes	Yes	Kadioglu et al., 2020
	Rutin	MD, SML	No	Yes	Kadioglu et al., 2020
	Lumacaftor	MD, SML	Yes	No	Chen Y. W. et al., 2020; Kadioglu et al., 2020
IL6, IL2, IL10, CASP3, IFNA1	Hydroxychloroquine, Ribavirin	PPIN	No	No	Kim and Kim, 2020
Angiotensin-Converting Enzyme 2	Isothymol	ADME;Drug-likeness	No	No	Abdelli et al., 2020

*MD, molecular docking; MDS, molecular dynamics simulations; HM, homology modeling; NBI, network-based identification; SML, supervised machine learning; DS: dynamics simulations; PPIN, protein–protein interaction network; PASS, prediction of activity spectra for substance; ADME, absorption, distribution, metabolism, and excretion*.

Different computational approaches or machine learning methods are being used in *in silico* drug screening. Among the widely used approaches, molecular docking and molecular dynamic simulation play the most crucial role in drug discovery and development process, which also figure importantly in the suggestion of drugs with potential against COVID-19 (Table 3). Molecular docking is a method that predicts the ideal location of one molecule to a second when bound to each other to form a stable complex. It is often used to predict the binding alignment of small molecule drug candidates to their protein targets in order to predict the affinity and activity of the small molecule (Guedes et al., 2014; Chaudhary and Mishra, 2016). Molecular dynamic simulation is a machine learning approach where predicted *in silico* structures or drug target compounds are usually exposed to virtual cellular environment for the analysis of the stability of *in silico* structures or docked compounds (Dong et al., 2013; Martinotti et al., 2020). Other computational approaches such as homology modeling (Krieger et al., 2003), network-based identification (Zhou et al., 2020), drug-likeness, or absorption, distribution, metabolism, and excretion (ADME) analysis (Butina et al., 2002) have been used extensively in the screening of extensive numbers of drug candidates against SARS-CoV-2 (Table 3).

SARS-CoV-2 is an enveloped positive-sense single-stranded RNA virus (ssRNA) consisting of 29,903 nucleotides and two untranslated sequences of 254 and 229 nucleotides at the 5′ and 3′ ends, respectively (Wu F. et al., 2020). These genes encode proteins responsible for the synthesis of surface spike glycoprotein, nucleocapsid phosphoprotein, envelope membrane glycoprotein, replicase complex, and five other proteins (Kadioglu et al., 2020). These proteins have been studied and suggested as potential drug targets which were also reported for many other corona viruses (Sanders et al., 2020). The mostly studied drug targets against COVID-19 include structural proteins of SARS-CoV-2 (spike glycoprotein, envelope protein, neucleocapsid protein), non-structural proteins of SARS-CoV-2 (Mpro, papain-like protease, RNA-dependent RNA polymerase, helicase), host cell target protein, and different cytokine release from host cellular environment (angiotensin-converting enzyme 2, transmembrane serine protease 2), and these are employed to both *in silico* and wet lab experiment for screening out effective inhibitors (Crosby et al., 2020; Li H. et al., 2020). In the present study, it has been found that Mpro (Chandel et al., 2020; Elmezayen et al., 2020; Kumar and Singh, 2020; Zhang D. et al., 2020), spike glycoprotein (S) (Hall and Ji, 2020; Kadioglu et al., 2020; Mohamed et al., 2020; Shah et al., 2020), nucleocapsid protein (Ge et al., 2020; Kadioglu et al., 2020; Musarrat et al., 2020; Sarma et al., 2020), and RNA-dependent RNA polymerase (RdRp) (Elfiky, 2020; Mohamed et al., 2020; Reiner et al., 2020) are the most widely studied drug targets for *in silico* drug development approaches, and several drug candidates suggested from computational screening are also being investigated under clinical trials (Table 3).

The review study recommended that Mpro is a widely targeted drug site for COVID-19 (Figure 1), and approximately 150 drug molecules have been suggested against Mpro of SARS-CoV-2 through different *in silico* drug repurposing techniques (Table 3). Lopinavir is the mostly suggested drug molecule for Mpro as recommended in recently published literature (Barros et al., 2020; Biembengut and de Arruda Campos Brasil de Souza, 2020; Chen Y. W. et al., 2020; Kumar and Singh, 2020; Mohamed et al., 2020; Mothay and Ramesh, 2020; Ortega et al., 2020; Pant et al., 2020; Shah et al., 2020). Besides, ritonavir (Barros et al., 2020; Chen Y. W. et al., 2020; Kumar and Singh, 2020; Mohamed et al., 2020; Mothay and Ramesh, 2020; Ortega et al., 2020; Pant et al., 2020; Shah et al., 2020), nelfinavir (Biembengut and de Arruda Campos Brasil de Souza, 2020; Chandel et al., 2020; Kumar and Singh, 2020; Mittal et al., 2020; Mohamed et al., 2020; Mothay and Ramesh, 2020), indinavir (Biembengut and de Arruda Campos Brasil de Souza, 2020; Hall and Ji, 2020; Mamidala et al., 2020; Mohamed et al., 2020; Shah et al., 2020), saquinavir (Barros et al., 2020; Biembengut and de Arruda Campos Brasil de Souza, 2020; Hall and Ji, 2020; Ortega et al., 2020; Shah et al., 2020), grazoprevir (Ibrahim et al., 2020; Kadioglu et al., 2020; Mohamed et al., 2020; Shah et al., 2020), darunavir (Mohamed et al., 2020; Ortega et al., 2020; Pant et al., 2020), HCQ (Barros et al., 2020; Mamidala et al., 2020; Srivastava et al., 2020), raltegravir, remdesivir, rosuvastatin, amprenavir, CQ, elbasvir, fluvastatin, oseltamivir, telaprevir, tenofovir, zanamivir, ivermectin, ledipasvir, rifabutin, teniposide, and velpatasvir have been forecasted as important drug candidates for blocking Mpro of SARS-CoV-2. Among these Mpro inhibitors, about 50 candidates are already Food and Drug Administration (FDA) approved (Home, 2013), and approximately 30 molecules are in different phases of clinical trials^1^.

Spike glycoprotein is the second most reported drug target for the treatment of COVID-19 (Figure 1). About 47 drug molecules have been claimed against spike glycoprotein. It is concluded that grazoprevir could be the most potent inhibitor of spike glycoprotein, as several studies are focusing on the efficiency of blocking spike by glycoprotein (Ibrahim et al., 2020; Kadioglu et al., 2020; Mohamed et al., 2020; Shah et al., 2020). Other therapeutics such as ivermectin (Ibrahim et al., 2020; Kadioglu et al., 2020), ledipasvir, teniposide, velpatasvir (Chen Y. W. et al., 2020; Kadioglu et al., 2020), and rifabutin (Bank et al., 2020; Kadioglu et al., 2020) also showed potential inhibitory action against the spike glycoprotein of SARS-CoV-2. Among the suggested candidate drugs of spike protein, 17 drug molecules were repurposed for COVID-19 and approved by the FDA (Home, 2013). About six of the candidate drugs of spike glycoprotein were investigated in clinical trials for the treatment of COVID-19^1^. In addition, a recently published study showed the value of certain motifs in the spike glycoprotein of the virus, most particularly a KRSFIEDLLFNKV motif by extending previous work on the application of the Q-UEL language and BioIngine to bioinformatics and precision medicine with genomics (Robson, 2020).

Nucleocapsid protein has also been extensively studied for screening out the effective therapeutic options for the ongoing pandemic. About 24 unlike drug molecules have been reported targeting nucleocapsid protein. Venetoclax is the most frequently reported drug substrate for this protein suggested by computational high throughput screening. Approximately 11 suggested drug molecules were FDA approved, and 9 clinical trials are ongoing for nucleocapsid inhibitors (Home, 2013; Chen Y. W. et al., 2020; Kadioglu et al., 2020). Another crucial drug target is RNA-dependent RNA polymerase (RdRp), which has also been evaluated by the machine learning computational approach to identify its potential blockers (Figure 1). The study revealed that favipiravir, remdesivir, and ribavirin could potentially be more potent inhibitors for RdRp (Elfiky, 2020; Mohamed et al., 2020; Reiner et al., 2020). Approximately six RdRp-targeted molecules were found in FDA yellow book, and six clinical trials were found to be evaluated for the safety and efficacy of newly predicted drug molecules (Elfiky, 2020; Mohamed et al., 2020; Pandit and Latha, 2020). Envelope protein of SARS-CoV-2 and 2′-o-ribose-methyltransferase are also important drug targets that have been described in different articles. Belachinal, macaflavanone E, and vibsanol B have been suggested against envelope protein using the molecular docking approach (Gupta et al., 2020). On the other hand, more than 20 drug candidates have already been suggested by *in silico* study against 2′-o-ribose-methyltransferase (Chen Y. W. et al., 2020; Kadioglu et al., 2020).

In addition, an N-terminal peptidase domain (PD) of ACE2 was found to react with the ectodomain of SARS-CoV-2 spike protein in the heart, lungs, kidneys, and intestine, and thus, ACE2 have also been found to be studied to target effective drug candidates (Figure 1) (Wrapp et al., 2020). Isothymol has been suggested against human ACE2 for the treatment of COVID-19, although wet lab investigation is yet to be started (Abdelli et al., 2020). Moreover, it has been found that HCQ has the maximum capacity to act on multiple drug targets, namely, Mpro, spike protein, RNA-dependent RNA polymerase, interleukin (IL) 6, IL2, IL10, and interferon alpha 1 (IFNA1) (Bank et al., 2020; Elfiky, 2020; Kim and Kim, 2020; Srivastava et al., 2020). Few other drug molecules were found to be effective against multiple drug targets. For examples, nelfinavir showed efficacy against Mpro and nucleocapsid protein (Chandel et al., 2020; Mittal et al., 2020; Mothay and Ramesh, 2020; Musarrat et al., 2020), whereas ivermectin was found to have potential against spike protein and 2′-o-ribose-methyltransferase (Chaudhary and Mishra, 2016; Mothay and Ramesh, 2020; Sharun et al., 2020). Grazoprevir reportedly exhibits inhibitory action against both spike protein and Mpro (Ibrahim et al., 2020; Kadioglu et al., 2020; Mohamed et al., 2020; Shah et al., 2020), whereas rifabutin was suggested against spike protein and nucleocapsid of SARS-CoV-2 (Bank et al., 2020; Kadioglu et al., 2020). About 215 clinical trial projects are ongoing in different phases on HCQ for the treatment of COVID-19 and studies leading to mixed conclusions for those candidate drugs (Gautret et al., 2020; Yazdany and Kim, 2020). There are a few other drugs such as ritonavir, lopinavir, chloroquine, remdesivir, ivermectin, oseltamivir, darunavir, and tenofovir currently being investigated in clinical trials for combating COVID-19^1^.

Recently, Jahan and Onay 2020 (Jahan and Onay, 2020) reviewed the antiviral potentials of various medicinal plants for inhibiting human coronaviruses. It also shows the importance of antiviral plants substances, particularly in the development of a broad spectrum medication for coronaviruses including SARS-CoV-2 responsible for COVID-19. Additionally, some other reports have been published on various immunoinformatics approaches. For example, a study aimed to formulate a multiepitope vaccine against SARS-CoV-2 by using the SARS-CoV-2 spike glycoprotein to determine the immunodominant T- and B-cell epitopes. They proposed a vaccine construct using four potential epitopes from each of the three epitope classes such as cytotoxic T lymphocytes, helper T lymphocyte, and linear B-lymphocyte epitopes (Samad et al., 2020). In addition, structural proteins (surface glycoprotein, envelope protein, and membrane glycoprotein) of SARS-CoV-2 were selected from GenBank, and several immunoinformatics coupled with computational approaches were employed to forecast B- and T-cell epitopes from the SARS-CoV-2 highly antigenic structural proteins to design an effective MESV (Tahir ul Qamar et al., 2020b). Another study to design a multiepitope vaccine, retrieving 27 reference sequences of SARS-CoV-2 proteins from the National Center for Biotechnology Information (NCBI) Protein Database (https://www.ncbi.nlm.nih.gov/protein), selecting proteins with ≥100aa and an antigenic score of ≥0.5 for further structural modeling (Dong R. et al., 2020).

## Concluding Remarks and Future Perspective

A successful vaccine would be the ultimate prophylaxis to defeat COVID-19, but no such vaccine is yet available for humans, and past experience suggests that the development of a new vaccine could take 4–28 years. For example, The New York Times estimated that a COVID-19 vaccine could be available in 2036, after completion of academic research, a series of preclinical and clinical trials, building factories, manufacturing, approval, and distribution (Thomson, 2020). Besides these, experimental vaccines cannot be injected on people without rigorous safety checks, which is extremely time consuming, as it involves numerous trial phases with many volunteers of different age groups, races, and health conditions, but such trials are critically important precursors to the approval of a new vaccine. Apart from all the frustration and despair, some of the vaccines that are recruiting volunteers and researchers conducting numerous trials have the potential to change the pandemic situation soon. Moreover, therapeutic drugs that have been approved against different viral infections previously might help in tackling COVID-19 if found to be effective in clinical trials. Besides the limitations, repositioning of those drugs and vaccines could ease the formulation, production, and distribution through established pharmaceutical supply chains to reach out to the market. As we see a rapidly growing amount of publications on COVID-19, it might help in finding an effective vaccine and the best practice for the management and treatment of COVID-19 symptomatic cases. Using bioinformatics tools for the prediction of epitopes to a higher level of accuracy in a shorter amount of time compared to traditional method will help us find a cure faster. This will help the scientific community for programming or investigating the set of rules for the detailed choice of amino acid residue sequences and their immunogenic potentiality toward designing vaccine candidates. This also opens up a possibility of building combinations with previous research by using pre-existing candidate therapies to speed the process of testing and discovery of effective pharmaceutical ways. Further research and scrutinizing may be warranted in the future to determine the benefits and optimal use of some of available treatment option through repurposing method. Besides vaccine and drug, complementary and substitute treatments using plant-based phytochemicals could be incredibly promising in the future for reducing the severity of infection. With all limitless possibilities in the near future, researches are still undergoing with several promising approaches with the final hope, the cure from COVID-19, and therefore controlling the pandemic worldwide. Nevertheless, most of the clinical trials registered in different websites are expected to complete within 2020 or early 2021, and consequently, it is hoped that effective prevention and treatment measures will see the light soon.

## Author Contributions

TS, MTH, MH, and MAH developed the initial concept. TS, MTH, SS, and CB wrote the abstract. TS, MTH, MAH, and WJ wrote the introduction. MTH, TS, and H-JK wrote the pathogenesis and symptoms of COVID-19. MTH, TS, WJ, and H-JK created Table 1 and led the section on vaccine. TS prepared the figures. TS, MAH, and AC created Table 2 and led the discussion on drugs of COVID-19. MH, EB, and MTH created Table 3 and wrote the *in silico* section. MH, TS, and MTH wrote the conclusion. SS, CB, and E-WL reviewed and corrected the whole manuscript. MAH, TS, and MTH formatted the manuscript for submission. All authors reviewed the final manuscript.

## Conflict of Interest

The authors declare that the research was conducted in the absence of any commercial or financial relationships that could be construed as a potential conflict of interest.

## Footnotes

^1^clinicaltrials.gov: U. S. National Library of Medicine.
